# Morel Production Associated with Soil Nitrogen-Fixing and Nitrifying Microorganisms

**DOI:** 10.3390/jof8030299

**Published:** 2022-03-14

**Authors:** Feng-Ming Yu, Ruvishika Shehali Jayawardena, Naritsada Thongklang, Meng-Lan Lv, Xue-Tai Zhu, Qi Zhao

**Affiliations:** 1 Key Laboratory for Plant Diversity and Biotechnology of East Asia, Yunnan Key Laboratory of Fungal Diversity and Green Development, Kunming Institute of Botany, Chinese Academy of Sciences, Kunming 650201, China; fm_yu2018@163.com; 2 Center of Excellence in Fungal Research, Mae Fah Luang University, Chiang Rai 57100, Thailand; ruvi.jaya@yahoo.com (R.S.J.); naritsada.t@gmail.com (N.T.); 3 School of Science, Mae Fah Luang University, Chiang Rai 57100, Thailand; 4 School of Chemical Engineering, Guizhou Institute of Technology, Guiyang 550003, China; lvmenglan@git.edu.cn; 5 College of Life Science, Northwest Normal University, Lanzhou 730070, China; zhuxuetai@nwnu.edu.cn

**Keywords:** soil microbes, network analysis, nitrogen fixation, nitrification

## Abstract

True morels (*Morchella*, *Pezizales*) cultivated in soil are subject to complex influences from soil microbial communities. To explore the characteristics of soil microbial communities on morel cultivation, and evaluate whether these microbes are related to morel production, we collected 23 soil samples from four counties in Sichuan and Yunnan Provinces, China. Based on ITS and 16S rDNA amplicon sequencing, the alpha diversity analysis indicated that the biodiversity of morel cultivation soil showed a downward trend compared with the bare soil. The results also showed that there were no significant differences in soil microbial communities between OC (bare soil) and OO (after one-year suspension of sowing). This means that, after about one year of stopping sowing, the component and structure of soil that once cultivated morel would be restored. In co-occurrence networks, some noteworthy bacterial microbes involved in nitrogen fixation and nitrification have been identified in soils with high morel yields, such as *Arthrobacter*, *Bradyhizobium*, *Devosia*, *Pseudarthrobacter*, *Pseudolabrys*, and *Nitrospira*. In contrast, in soils with low or no morel yield, some pathogenic fungi accounted for a high proportion, including *Gibberella*, *Microidium*, *Penicillium*, *Sarocladium*, *Streptomyces*, and *Trichoderma*. This study provided valuable information for the isolation and culturing of some beneficial microbes for morel cultivation in further study and, potentially, to harness the power of the microbiome to improve morel production and health.

## 1. Introduction

True morels (*Morchella* spp., *Morchellaceae*, *Pezizales*), as highly prized edible mushrooms, are of great economic and scientific value [1,2]. They are distinguished by their unique honeycomb-like appearance of hollow fruiting bodies and are widely distributed in temperate regions of the Northern Hemisphere [1,2]. Wild morels are enigmatic and ephemeral because they usually only bear fruit for a few weeks every spring, except for some autumnal species [2,3,4]. For centuries, the fruiting bodies of morel have been used as food and, recently, it has been proved to be beneficial to health, especially in antibacterial, antioxidant, and anti-inflammatory properties [5,6]. These important qualities not only propel morel into a popular edible mushroom, but also lead to the increase in market demand and scarcity of wild resources. Given the above, the artificial cultivation industry of morel in Asia, especially in China, has been growing rapidly, which is helpful to alleviate the market pressure [1,2].

Artificial cultivation of morel has been the focus of global research for more than 100 years, beginning with the first report in 1882 on outdoor cultivation in France [7]. The early indoor cultivation of *M. rufobrunnea* was traced back to the 1980s in the United States [8]. In recent years, with the improvement and wide application of exogenous nutrient bags, seven morel species (i.e., *M. sextelata*, *M. eximia*, *M. exuberans*, *M. importuna*, *M. owneri*, *M. rufobrunnea*, and *M. tomentosa*) and two phylogenetic species (i.e., *Mel-13* and *Mel-21*) have been successfully cultivated. *Morchella sextelata*, *M. eximia*, and *M. importuna* are the main cultivated varieties in China [1,9,10,11]. From 2011 to 2020, the cultivated area rapidly expanded from 200 ha to 10,000 ha, and the yield of fresh morel increased from less than 750 kg/ha to 15,000 kg/ha [2,12]. The annual yield in 2020 upped to 15,000 tons (fresh weight) in China and maintained its growth trend (unpublished data). However, a field survey of morel cultivation from 2019 to 2020 found that half of the morels’ cultivation failed to bear fruit or exhibited a low reproduction rate, and more than 70% of growers could not obtain stable profits [2,13]. Unsuccessful cases of morel culture may be due to the unstable quality of strains (e.g., high genetic diversity, spawn aging, and degeneration) [9,14,15], pathogens [16,17,18,19], environmental factors (e.g., temperature, humidity, and soil moisture) [20,21], exogenous nutrition [22], soil characteristics, and soil microbial community dynamics [13,23,24,25,26,27].

Recent studies have shown that soil-associated microbes are known to influence the growth and development of some mushrooms that need a casing of soil layer, e.g., *Agaricus bisporus* [28,29], *Ganoderma lucidum* [30,31], *Phlebopus portentosus* [32], and *Stropharia rugosoannulata* [33]. The roles of these soil-associated bacteria and fungi include the prevention of pathogenic infections, transport and cycling of materials in the soil for mushroom growth, and the induction of primordial fruiting body formation [24,34,35,36]. Beneficial bacteria *Pseudomonas* has been reported as being associated with the sclerotia formation, primordia formation, and fruiting bodies in some cultivated species [37] of *Agaricus* [38], *Morchella* [36], and *Pleurotus* [39,40]. As far as commercial cultivation of morel is concerned, farmland and forest farming are the main cultivation patterns in China at present [10]. Considering that morel reproduction is inseparable from soil, and covering soil is a key step in the sowing process, some researchers have speculated that certain soil substances play a crucial role in morel cultivation [13,25,26,27].

Most research on dynamics of the microbial community associated with wild and cultivated morel have been conducted by means of ribosomal amplicon-based approaches. Orlofsky and colleagues (2021) revealed the ecological progression of the bacteria community associated with *M. rufobrunnea* fruiting in natural habitat [41]. Its progression began with the establishment of photoautotrophic N-fixing bacterial mat on bare soil; then, pioneer heterotrophic bacteria in soil under young morel became dominant; finally, under mature fruiting body, the bacterial population changed to saprobes, organic-N degraders, denitrifiers, insect endosymbionts, and fungal antagonists [41]. For cultivated morel, based on ITS and 16S rDNA high-throughput amplicon sequencing data, Longley and colleagues (2019) determined the microbial community changes in the key stages of morel fructification. The results showed trays with successful fruiting were dominated by *Gilmaniella* and trays that failed to fruit were dominated by *Cephalotrichum*. They also found that the fungi *Gilmaniella* and bacteria *Bacillus* were abundant in substrates supporting *M. rufobrunnea* fruiting indoors [25]. In the same year, Benucci and colleagues (2019) reported that *Pedobacter*, *Pseudomonas*, *Stenotrophomonas*, and *Flavobacterium* formed the core microbiome of *M. sextelata* ascocarps in outdoor greenhouses [26]. Later, Tan and colleagues (2021) demonstrated that the soil physicochemical characteristics may not be the main reason for the difference between the successful fructification and nonfructification of morel farms in Chengdu Plain, China. Their findings also showed that the morel yield was positively correlated with the alpha-diversity of fungal communities, and that community evenness contributed to the higher diversity of the successfully fructified soils [13].

Indeed, growing morels requires an understanding of environmental microorganisms, especially those living in soil. Previous studies on microbial communities related to the morel cultivation were based on a single region or farm, and the in-depth research on soil communities of morel in different places was still scarce. Considering the regional differences of soils, large-scale sampling of morel cultivation soils will facilitate an exhaustive exploration of the relationship between morel and soil microorganisms. It was hypothesized that microbiome diversity, abundance, and specific microbiomes might play an important role that influences the morel fructification. To better comprehend the interaction between cultivated morels and their surrounding soil microbiome, this study employed high-throughput amplicon sequencing to investigate the diversity and structure of soil fungal and bacterial community in morel-cultivating areas. To reveal differences in soil microbial abundance and structure after morel cultivation, 15 soil samples from three sites were collected in Songming city, Yunnan, and Guanghan city, Sichuan. Meanwhile, in order to assess whether the soil microbial community is related to the morel yield and report the indicative soil fungal and bacterial communities related to the morel fructification, an additional eight soil samples (i.e., SM0, SM1, SM2, SM3, WD1, WD2, WD3, and DY1) were taken for in-depth analyses.

## 2. Materials and Methods

### 2.1. Soil Sample Collection

**Figure 1:**
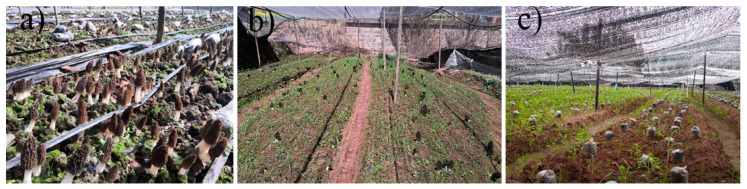
Soil samples of different morel production. Take site 1 in Songming as an example, (**a**) soil samples with high morel yield, (**b**) soil samples with low morel yield, (**c**) soil samples without morel yield.

To investigate the changes in soil microbial community after cultivating morel and the characteristics of soil microbial community associated with morel productivity, soil samples were collected from four representative plantations in two provinces of China (Table 1 and Figure 1) and, in total, 20 soil samples with diverse morel yield and three blank soil samples were collected for amplicon-based microbial community analyses. The protocol used for soil sampling in this study was as follows: to minimize the effects of soil spatial variability, samples were taken using a “W”-shaped path for each plot and soil samples were collected to a depth of 3–5 cm using a shovel; each plot consisted of nine individual soil cores, which were then mixed; after thoroughly mixing, the last four replicates were obtained by using quartering [13,33]. In total, 92 samples (23 plots × 4 replicates) were collected to be analyzed. The collected fresh soil was sieved (<2 mm) to remove stones and plant roots [33]. All soil samples were placed in 50 mL sterile centrifuge tubes and stored at −80 °C for DNA extraction. To ensure the sterility of collection tubes, empty tubes were used for negative control.

In this study, MUCS = control soil, the soil samples without morel cultivation located in a nearby farm (the distance < 50 m), and MCS = the soil samples with morel cultivation. Briefly, a total of 23 soil samples were treated in three groups (Table 1): group I consisted of NC, NF, NN, NSN, OC, OO, OCP, OCF, ORF, GHC, GH1, GH2, GH3, GH4, and GH5; group II was made up of NF, OCP, OCF, ORF, GH4, GH5, SM1, SM2, SM3, WD1, WD2, and WD3, representing soil of samples with a high yield of morels (≥1500 kg/ha); and group III was composed of NN, NSN, GH1, GH2, GH3, SM0, and DY1, representing soil of samples with low or no morel productivity (<450 kg/ha).

### 2.2. DNA Extraction and Sequencing

Total soil DNA was extracted from 0.5 g fresh soil by the MoBioPowerSoil™DNA Isolation Kit (Mo Bio Laboratories Inc., Carlsbad, CA, USA) according to the manufacturer’s protocol. DNA concentration and purity were measured using a Nanodrop NC2000 spectrophotometer (Thermo Scientific, Wilmington, DE, USA). For site 1 and site 2, 16S and ITS2 amplicon library preparation and sequencing were performed according to the manufacturer’s protocol at BGI-Wuhan, China. The primers 341F (ACTCCTACGGGAGGCAGCAG) and 806R (GGACTACHVGGGTWTCTAAT) were used to amplify the V3–V4 region of the bacterial 16S rRNA gene. The primers ITS3 (GCATCGATGAAGAACGCAGC) and ITS4 (TCCTCCGCTTATTGATATGC) were used to amplify the internal transcribed spacer (ITS) region of fungi. For sites 3–6, 16S (V3–V4 region, 338F: ACTCCTACGGGAGGCAGCA; 806R: GGACTACHVGGGTWTCTAAT) and ITS (ITS1 region, ITS5F: GGAAGTAAAAGTCGTAACAAGG; ITS1R: GCTGCGTTCTTCATCGATGC) amplicon library preparation and sequencing were performed at Personal Biotechnology Co., Ltd., China. The purified PCR products were sequenced, and then amplicon libraries were prepared using the Illumina MiSeq platform using the MisSeq Reagent Kit V3 (600 cycles). Raw reads were processed using the QIIME 1 software package [42]. Briefly, based on the sequence lengths ≥ 160 bp and without fuzzy base N, the sequences with a mismatched base number > 1 of 5′ end primer and with >8 identical consecutive bases were discarded, and then the chimera sequences were filtered by the USEARCH v5.2.236 algorithm [42,43,44]. After quality control, quantification, and normalization of the DNA libraries, the obtained high-quality sequences were merged as operational taxonomic units (OTUs) using UCLUST at a 97% similarity threshold cutoff [45]. To process the taxonomic classification of OTUs, the representative sequences of each OTU were generated and aligned against the Greengenes database [46], RDP databases [47], and Sliva database (Sliva_V132) [48] for bacterial OTUs and UNITE database [49] for fungal OTUs, respectively. The relative abundance data for taxa were generated based on the read count for each taxon across samples by using the total-sum scaling method. Rare (OTUs with an abundance less than 0.001% of the total sequences of all samples [50]), nonfungal, and nonbacterial OTUs were removed from final analysis in this study. All raw sequence data were deposited in National Center for Biotechnology Information (NCBI) database with BioProject accession number PRJNA813979.

### 2.3. Statistical Analyses

Alpha-diversity analyses (within the community), including ACE [51] and Chao 1 [52], and Simpson [53] and Shannon [54] diversity indices were analyzed at the OTU level using QIIME. Beta-diversity (principal component analysis, PCA) analyses were performed for the observed samples [55]. The statistical significance of the difference between the means of samples was determined by one-way ANOVA (Team, 2015). To detect differential taxa of different samples, pairwise comparisons of the number of sequences (absolute abundance) among taxa in genera level were performed using Mothur with Metastats [56].

To explore the co-occurrence patterns between soil microbial taxa in group II (high-yield group) and group III (low- and no-yield group), the co-occurrence network was performed using the Spearman correlation matrix [57]. Fungal genera and bacterial genera with the top 50 relative abundances in each group were selected for analysis. Networks were calculated by all possible pairwise Spearman’s rank correlations between the microbial genera with an absolute cut-off *r*-value of 0.6. After applying the Benjamini–Hochberg’s false discovery rate correction [58], the edges with merged *p*-values below 0.05 were retained to improve network precision. The robust correlations selected from pairwise comparisons of the genera abundance form a correlation network, where each node represented one genus, and each edge represented a strong and significant correlation between nodes [59]. The network structure was visualized and analyzed using Cytoscape, and related parameters (average clustering coefficient, average path length, and modularity) were calculated [59,60].

## 3. Results

### 3.1. Sequencing Results

Approximately 424,666 and 32,313 OTUs were generated for the 16S and ITS sequencing samples, respectively. After filtration, a total of 4036 fungal community OTUs with a read length of predominantly 200–350 bp, and 6029 bacterial community OTUs with a read length of mainly 400–450 bp were obtained. Rarefaction analysis was conducted on each sample, and the microbial rarefaction curves of all samples gradually exhibited a gentle trend, which implied that the sequenced depths were saturated to reflect the diversity of the samples.

### 3.2. Microbial Community Composition and Structure Analysis in All Soil Samples

**Figure 2:**
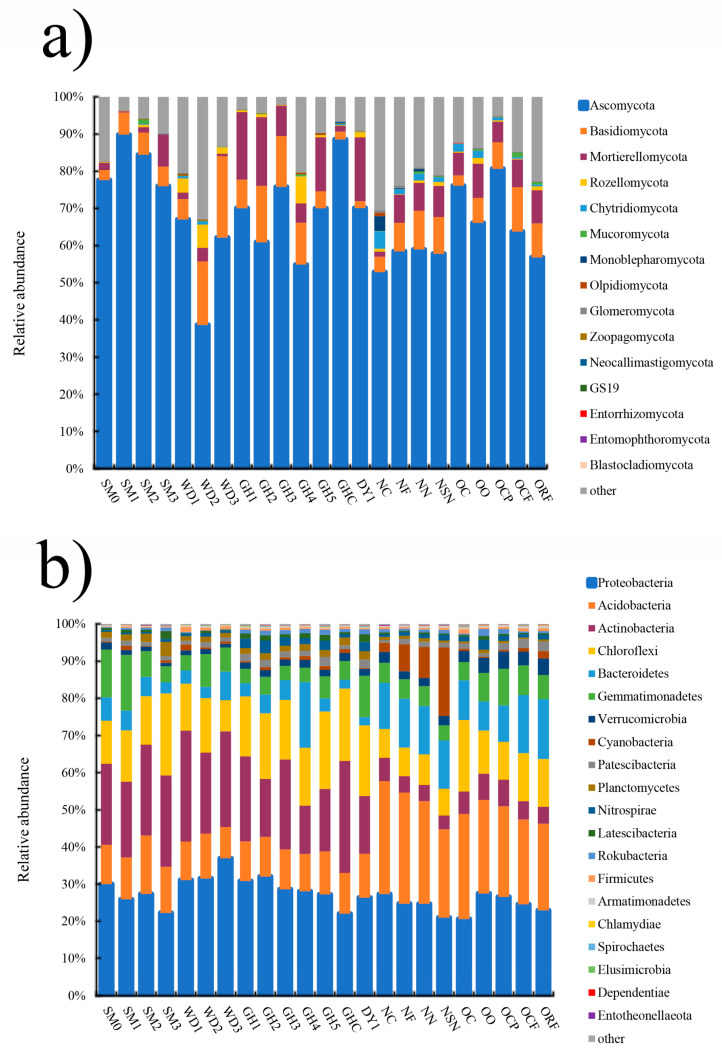
Microbial community composition and abundance distribution in all soil samples. (**a**) Fungal phyla-level distributions based on ITS amplicon data. (**b**) Bacterial phyla-level distributions based on 16S amplicon data.

Fungal communities of all soil samples were successively dominated by *Ascomycota*, *Basidiomycota*, *Mortierellomycota*, *Rozellomycota*, *Chytridiomycota*, *Mucoromycota*, and *Monoblepharomycota*. Percentages of *Ascomycota* and *Basidiomycota* were ranged from 38.641% to 89.831% and 1.843% to 21.915%, respectively (Figure 2a). The dominant bacterial phyla were *Proteobacteria*, *Acidobacteria*, *Actinobacteria*, *Chloroflexi*, *Bacteroidetes*, *Gemmatimonadetes*, *Verrucomicrobia*, *Cyanobacteria*, the *Patescibacteria* group, *Planctomycetes*, *Nitrospirae*, the *Latescibacteria* group, the *Rokubacteria* group, and *Firmicutes* (Figure 2b).

**Figure 3:**
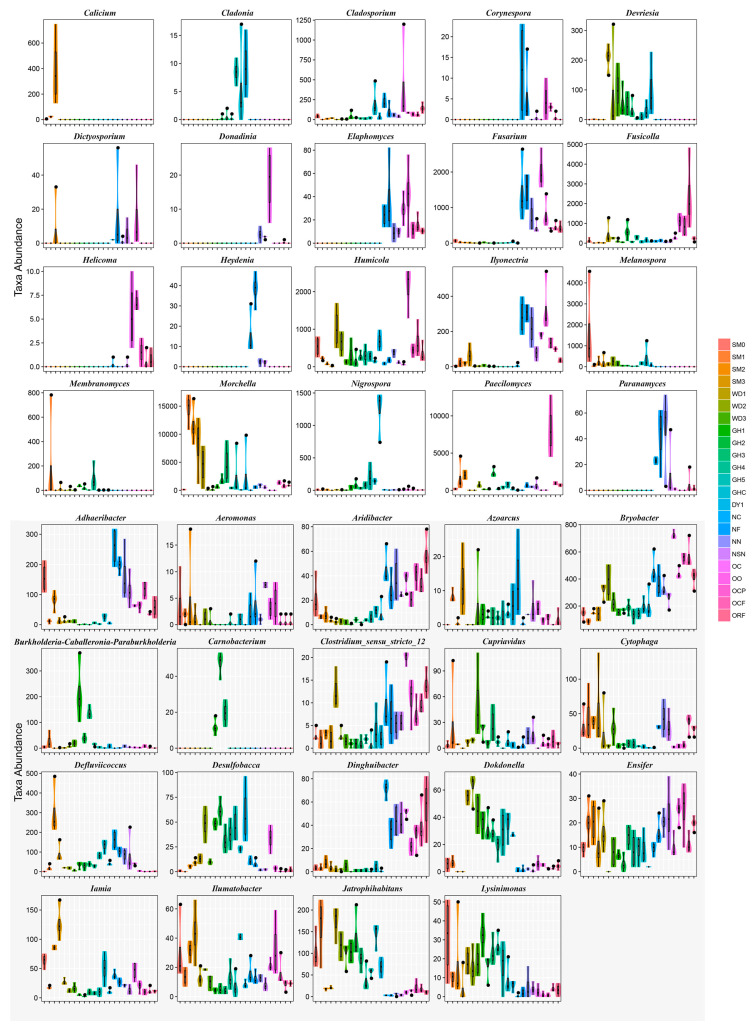
Metastats analysis in all soil samples. The abscissa is the first 39 taxa with the most significant difference, and the ordinate is the sequence quantity of each taxon in each sample. The “fat and thin” of “violin” reflects the level of density of sample data distribution (the wider width indicates more samples corresponding to this sequence quantity).

The extent of the top 20 fungal species-specific variations at the genus level (Metastats analysis, Figure 3) revealed the predominance of *Corynespora*, *Dictyosporium*, *Elaphomyces*, *Fusarium*, *Heydenia*, and *Paranamyces* in site 1; *Cladosporium*, *Donadinia*, *Fusarium*, *Fusicolla*, *Helicoma*, *Humicola*, *Hyonectria*, and *Paecilomyces* in site 2; *Cladonia* in site 3; *Calicium*, *Dictyosporium*, *Melanospora*, *Membranomyces*, and *Morchella* in site 4; *Devriesia* in site 5; and *Nigrospora* in site 6. Considering the soil samples related to the morel production, *Paranamyces*, *Melanospora*, and *Nigrospora* with high abundance were detected in soil samples related to low- and no- morel yield; whereas *Corynespora*, *Dictyosporium*, *Elaphomyces*, *Heydenia*, *Paecilomyces*, *Fusicolla*, *Calicium*, *Devriesia*, *Melanospora*, *Membranomyces*, and *Morchella* were most abundant in soil related with high morel yield. For 19 bacterial genera, Metastats analysis showed *Burkholderia*–*Caballeronia*–*Paraburkholderia* and *Carnobacterium* seemed to be biomarkers in site 3.

### 3.3. The Beta-Diversity of Soil Microbial Communities in All Samples

**Figure 4:**
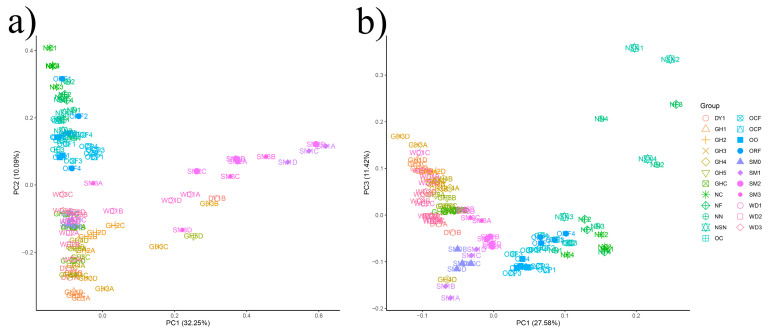
PCA of the microbial communities for fungal communities (**a**) and bacterial communities (**b**) in all soil samples.

To compare the beta-diversity of soil microbial communities, PCA was performed on the ITS (Figure 4a) and 16S rDNA (Figure 4b) data. For the fungal communities, the variance of the first axis (32.25%) was due to differences in fungal communities between NC and SM1, and variation obtained for the second axis (10.09%) was contributed to by the differences between NC and GH1. For the bacterial communities, their differences between GH3 and NF explained the variance obtained in the first axis (27.58%), while the variance in the second axis (11.42%) was mainly due to the differences within NSN. PERMANOVA analyses showed that 0.831 and 0.693 of the sum of squares variation were explained by fungal and bacterial communities, respectively (Table 2). The distinction of fungal communities appeared to be more pronounced than that of bacterial communities.

### 3.4. Heatmap Analyses of Soil Microbial Communities

**Figure 5:**
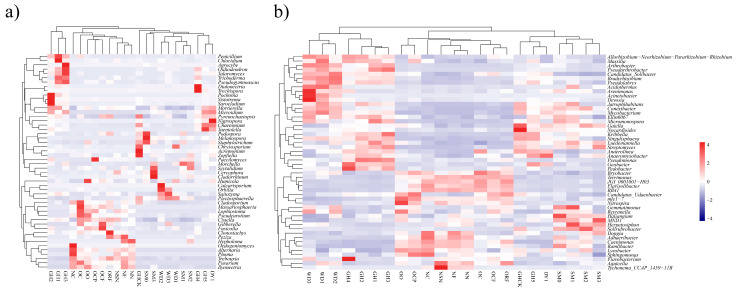
Heatmap of the relative abundances of top 50 fungal genera (**a**) and top 50 bacterial genera (**b**). Gradient color from blue to red represents a relative abundance of each taxon from low to high. Scale, the relative abundance of genus at row normalization by removing the mean (centering) and dividing by the standard deviation (scaling).

The top 50 fungal genera and top 50 bacterial genera were selected for heatmap analyses, respectively, which indicated that the dominant genera varied greatly among different soil samples (Figure 5). Amongst fungal genera (Figure 5a), there were significant differences in the relative abundance of *Morchella* in MCS, even though the standardized initial sowing rate of morel was performed. In MCS with high yield, the relative abundance of *Morchella* ranged from 0.52% (WD2) to 62.13% (SM1). However, the relative abundance of *Morchella* in GH3 and DY1 that showed low or no yield incredibly hit 19.84% and 11.50%, respectively. In GH2 where morel fruiting bodies were infected by some diseases, the relative abundance of *Morchella* was 7.69%. Those cases indicated the relative abundance of *Morchella* in the soil was not the main reason for its successful fructification. From the heatmap analysis of bacteria (Figure 5b), crop-beneficial microbes *Bradyrhizobium* (in WD2, 2.48%), *Devosia* (in WD3, 1.50%), *Flavobacterium* (in GH4, 9.96%), *Pedobacter* (in GH4, 5.25%), and *Pseudomonas* (in GH2, 4.14%) showed higher proportions. Amongst those genera, the relative abundance of “beneficial bacteria” *Pseudomonas* ranged from 0.06% (DY1) to 4.14% (GH2).

### 3.5. Changes in Alpha-Diversity on Soil Microbial Community between MUCS and MCS

**Figure 6:**
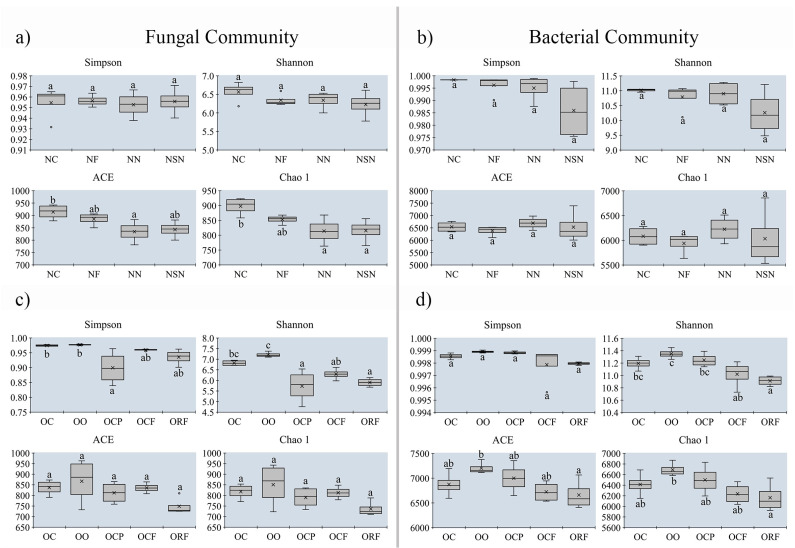

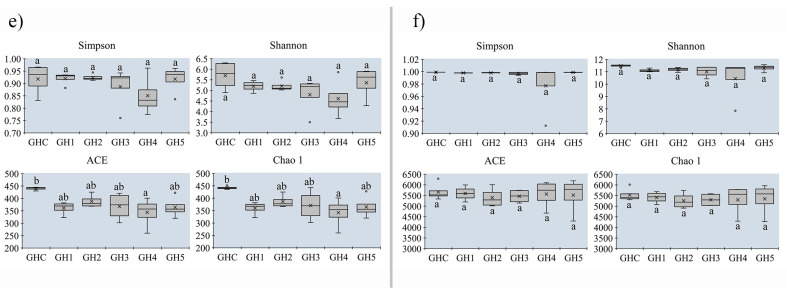
Alpha-diversity estimators of the soil fungal (**a**,**c**,**e**) and bacterial communities (**b**,**d**,**f**) of the investigated morel farms. For individual index boxes, different letters indicate significant differences at *p* = 0.05 (ANOVA) between means (Tukey’s HSD pairwise comparisons, n = 4).

To investigate the effect of morel cultivation on soil microbial communities, group I (containing sites 1–3) was selected for further study (Figure 6). For fungal communities collected from sites 1 and 3, the richness indices (ACE and Chao 1) were statistically higher in MUCS than in MCS (*p* < 0.05), while the Simpson and Shannon indices did not differ among all soil samples. In site 2, the diversity indices of fungal community (Simpson and Shannon) in OC and OO were significantly higher than in OCP, OCF, and ORF (*p* < 0.05). For bacteria, the taxonomic diversity (Simpson and Shannon) and richness (ACE and Chao 1) had no statistical difference in all soil samples collected from sites 1 and 3. In site 2, the taxonomic richness (ACE and Chao 1) and diversity (Shannon) indices were statistically higher in MUCS than in MCS, while there were no differences for Simpson indices. Those results indicated that the component and structure of soil microbes have changed to varying degrees after cultivation of morel, and compared with MUCS, the taxonomic richness and diversity indices of MCS showed a decreasing trend. However, the alpha-diversity estimators of the soil communities showed surprisingly no correlation with the output level of morels.

Remarkably, the alpha-diversity indices had no significant difference between OC and OO, which showed, after a year of cessation cultivation, the diversity and richness in the soil where morels have been cultivated may be expected to recover (Figure 6c,d).

### 3.6. Co-Occurrence Patterns of Microbial Communities Associated with Morel Production

To investigate differences in the co-occurrence patterns related to morel production, tweleve high-yield soil samples (group II: SM1, SM2, SM3, WD1, WD2, WD3, GH4, GH5, NF, OCP, OCF, and ORF) and seven low- or no-yield soil samples (group III: SM0, GH1, GH2, GH3, DY1, NN, and NSN) were selected for network analysis, respectively.

**Figure 7:**
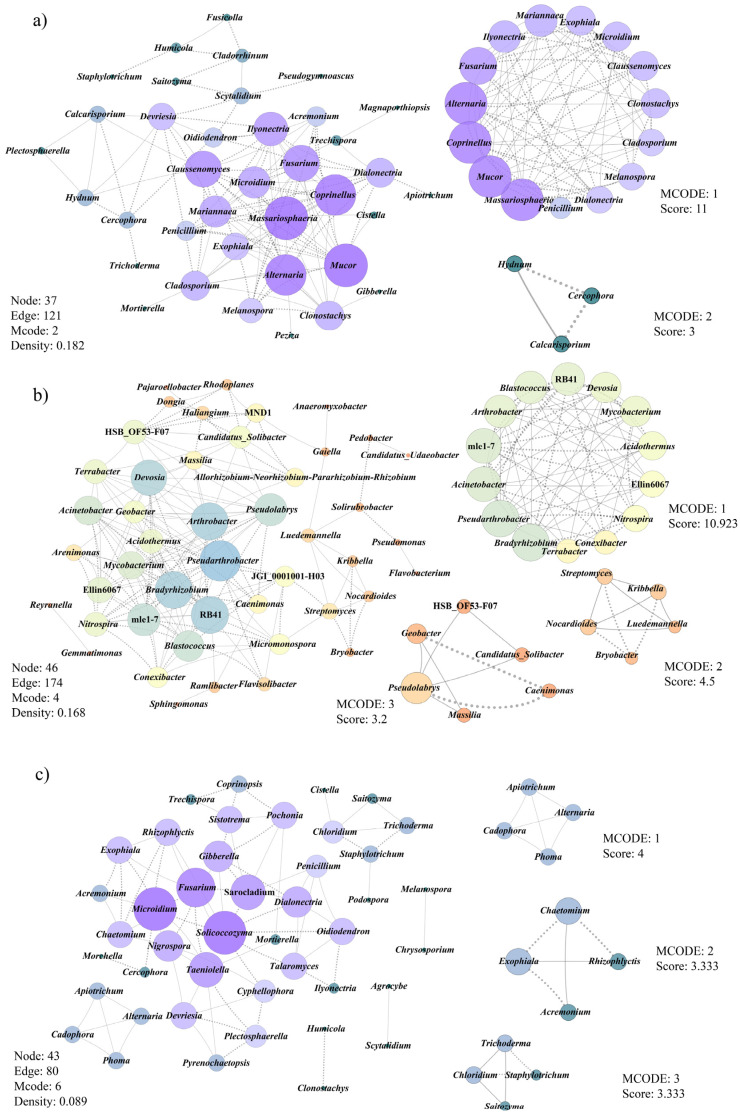

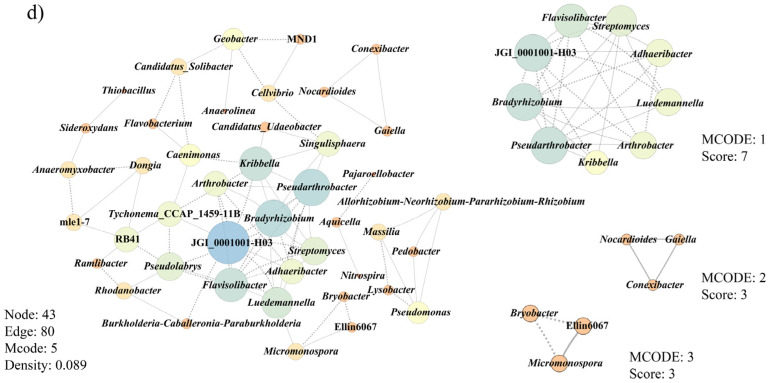
Microbial co-occurrence network in MCS associated with the high-yield group ((**a**) fungi, (**b**) bacteria) and low- or no-yield group ((**c**) fungi, (**d**) bacteria). The solid line represented positive co-occurrence and the dotted line indicated negative co-occurrences. Node size indicates the number of taxa connected to it (node size represents the degree of each taxonomy), the size from small to big represents the degree from low to high.

In group II, the fungal microbial network was composed of 37 nodes and 121 edges, with an average number of neighbors of 6.54 (Figure 7a), whereas the network for bacterial microbes had 46 nodes and 174 edges, with an average number of neighbors of 7.57 (Figure 7b). The highest degrees of association were detected in fungal genera named *Massariosphaeria* (degree = 16) and *Mucor* (degree = 16), followed by *Alternaria* (degree = 15) and *Coprinellus* (degree = 15). Within the bacterial communities, 15 bacterial genera (15/46) were detected in the co-occurrence network with a degree ≥ 10, of which *Pseudarthrobacter* (degree = 20) was the highest degree, followed by *Arthrobacter* (degree = 18), *Bradyhizobium* (degree = 18), and RB41 (degree = 18). Amongst those bacterial genera, *Arthrobacter*, *Bradyhizobium*, *Devosia*, *Pseudarthrobacter*, *Pseudolabrys*, and *Nitrospira* proved to play an important role in the nitrogen cycle. This indirectly indicates that the yield of morel may be positively correlated with these microbes involved in the nitrogen cycle.

In group III, two fungal genera *Microidium* and *Solicoccozyma* and a single bacterial genera *JGI_0001001-H03* had the highest degrees of association (9 and 11, respectively). Analysis of the microbial network properties, both fungal and bacterial communities revealed higher densities in the high-yield network than in the low- or no-yield network. Some common pathogenic fungi, such as *Gibberella*, *Microidium*, *Penicillium*, *Sarocladium* and *Streptomyces*, and *Trichoderma* were present in the co-occurrence network related to low or no yield. Genus *Cercophora* showed a negative relationship with *Morchella*. Unexpected high relative abundance of those pathogenic microbiomes in group III may lead to the decline in morel yield and serious diseases. Collectively, potential pathogenic genera *Microidium* and *Fusarium* were found in both group II and group III. After modularizing the nodes in the network, two fungal modules and top three bacterial modules of group II, and both top three fungal and bacterial modules of group III were visualized in Figure 7, with network scores ranging between 3 and 11.

## 4. Discussion

Under current cultivation techniques, covering soil is crucial for morel development [10]. The rhizosphere soil harbors diverse microbes, most of which undeniably benefit crops by preventing pathogenic infection and transferring nutrients from the soil [34]. Thus, it is important to understand the taxonomic, interaction, and functional components of the rhizosphere microbiome for sustainable morel production.

### 4.1. Microbial Community Dynamics during Morel Cultivation

Cultivating morel could affect the component and structure of the soil microbiome. Compared with morel uncultivated soil (MUCS), the diversity and richness of fungi and bacteria in the morel cultivated soil (MCS) have declined to varying degrees. In site 2, the soil sample OO was taken from a farm where the morel cultivation had been stopped for one year. The α-diversity analyses showed that there was no significant difference in microbial diversity and abundance between OC and OO, and PCA analyses also showed that microbial communities in OC and OO were similar.

In field-based and semi-artificial cultivation, continuous cropping obstacles are often found in cultivating several kinds of mushrooms, such as in *Agaricus bisporus*, *Ganoderma lucidum*, *Morchella* spp., *Phallus indusiatus*, *Pleurotus eryngii*, and *Stropharia rugosoannulata*. Previous studies indicated that the change in soil microbiome was the cause of continuous cropping obstacles [61]. Continuous cropping in morel cultivation could reduce yield, quality of fruiting bodies, and disease resistance. At present, the continuous cropping obstacles have not been effectively solved. With the extension of planting time and the increase in continuous cropping land, continuous cropping obstacles will restrict the development of morel industry. In this paper, α-diversity and PCA analyses implied that the soil cultivated with morel can recover to the fungal and bacterial community status of the original soil after one year. However, because the physicochemical properties of soil samples and the microbial metabolites were not analyzed, whether intermittent morel cultivation can alleviate continuous cropping disorders still needs further research.

### 4.2. Positive Effects of Nitrogen Cycling on High Yields of Morels

In group II, *Arthrobacter*, *Bradyhizobium*, *Devosia*, *Pseudarthrobacter*, *Pseudolabrys*, and *Nitrospira* in the center of the network proved to play an important part in the nitrogen cycle (Figure 7b). Members in *Pseudarthrobacter* showed a positive correlation with the content of available nitrogen in various agricultural ecological soil systems, confirming that *Pseudarthrobacter* could promote crop growth by nitrogen fixation [62,63]. Based on the genome data, Buckley [64] indicated that *Pseudarthrobacter* and *Arthrobacter* were able to reduce nitrate to nitrite. *Bradyrhizobium*, *Devosia*, and *Pseudolabrys* were also proved to have nitrogen fixation and nitrification, which played vital roles in agricultural productivity [24,65,66,67]. In addition, comammox *Nitrospira* members can complete oxidation of ammonium to nitrate for the nitrification [68].

Morels and truffles are very precious edible mushrooms in ascomycetes. Barbieri and colleagues (2010) reported nitrogen fixation by *Bradyrhizobium* cells occurred within *Tuber magnatum* ascocarps and it was the first time the nitrogenase expression gene and activity within truffle was demonstrated [65]. Using high-throughput sequencing combined tissue culture, Chen and colleagues (2019) reported the bacterial communities of Chinese black truffle, *Tuber indicum*, from different geographical regions of China. Their findings revealed *Bradyrhizobium* was the dominant genus in fruiting bodies of *T. indicum*, and they suspected that some cultural bacteria strains that exhibited the potential abilities of nitrogen fixation and inorganic phosphorus solubilization may play an important role in truffle seedling growth, fruiting body development, and even truffle evolution process [69]. In the natural habitat of *M. rufobrunnea*, the establishment of the photoautotrophic nitrogen-fixing microbial pad and heterotrophic nitrification were two important ecological processes for morel fruiting [41]. For the cultured morels, Tan and colleagues (2021) showed that nitrogen nutrients in the substratum were essential for morel fruiting, and the appropriate form of nitrogenous substances was requested [70]. Across the whole fruiting process of morels, they demonstrated that the C1 substratum, with the highest morel yield, maintained more abundant nitrate N; in comparison, the C2 substratum (a lower yield of morel fructification) harbored higher levels of ammonium N than nitrate N, which indicated that nitrate N had a positive effect on the morel fructification [70]. Previously reports also showed that ammonia accumulation in *A. bisporus* substratum can adversely affect mycelium growth while favoring fungal parasites [34]. In this study, it was found that the groups involved in nitrogen fixation and nitrification were placed in hubs in the co-occurrence network related to high yield of morel. Combined with previous studies, nitrogen nutrients and nitrogenous form were of great significance to morel fructification. Therefore, the following work should be focused on the interaction between morels and some microbiomes that have the ability of nitrogen fixation and nitrification.

### 4.3. Pathogenic Fungi Affect Morel Fructification

In morel cultivation, fungal diseases threaten the morel production and cause economic losses. In group III, the relative abundance of those pathogenic fungi, such as *Gibberella*, *Microidium*, *Penicillium*, *Sarocladium* and *Streptomyces*, and *Trichoderma*, may be the main cause of low or no yield of morels. At present, *Cladobotryum* spp., *Diploöspora longispora*, *Fusarium* spp., and *Paecilomyces penicillatus* were considered as the four most serious disease-causing fungi of the morel industry [16,17,18,19,71]. However, how many potential pathogenic fungi are there? What is the pathogenicity of these fungi to morel? Further, how can they be identified and controlled? These problems still need further in-depth research.

Based on a large-scale investigation of morel farms, this study revealed the effect of cultivating morel on soil microbial community and identified the soil microbial cortege associated with morel yield. The results showed nitrogen-fixing and nitrifying microorganisms may promote morel fructification, while pathogenic fungi may seriously affect morel yield. In the future, the relationship between morels and the participation in nitrogen fixing and nitrification needs to be further studied. Meanwhile, the identification, prevention, and control of potentially pathogenic fungi are urgent issues to be solved. This study provided valuable information for understanding the interaction between morel production and soil fungal as well as bacterial communities, and provided useful microbial resources for morel cultivation in future.

## Figures and Tables

**Table 1:** Information of soil samples in this study.

Site	Code of Soil Sample	Detail in Morel Production	Group I	Group II	Group III	Location
1	NC	Control soil	MUCS			Songming, Yunnan
	NF	high yield	MCS	H		
	NN	low yield	MCS		L	
	NSN	no yield	MCS		N	
2	OC	Control	MUCS			Songming, Yunnan
	OO	Stop cultivating morels for a year	MUSC			
	OCP	high yield	MCS	H		
	OCF	high yield	MCS	H		
	ORF	high yield	MCS	H		
3	GHC	Control	MUCS			Guanghan, Sichuan
	GH1	no yield	MCS		N	
	GH2	low yield with disease	MCS		L	
	GH3	no yield	MCS		N	
	GH4	high yield	MCS	H		
	GH5	high yield	MCS	H		
4	SM0	no yield			N	Songming, Yunnan
	SM1	high yield		H		
	SM2	high yield		H		
	SM3	high yield		H		
5	WD1	high yield		H		Wuding, Yunnan
	WD2	high yield		H		
	WD3	high yield		H		
6	DY1	no yield			N	Deyang, Sichuan

**Table 2:** Permutational multivariate analysis of variance (PERMANOVA) results for both fungal and bacterial communities associated with morel cultivation soil. Permutations = 999. Significant *p*-values at *p* ≤ 0.05 are highlighted in bold.

Factor	Fungal Community				Bacterial Community			
	Df	F. Model	R^2^	*p*	Df	F. Model	R^2^	*p*
Soil samples	22	15.414	0.831	0.001	22	7.088	0.693	0.001
Residuals	69		0.169		69		0.307	
Total	91		1.000		91		1.000	

## Data Availability

All raw sequence data have been deposited to NCBI with BioProject accession number PRJNA813979.
